# Metformin directly acts on mitochondria to alter cellular bioenergetics

**DOI:** 10.1186/2049-3002-2-12

**Published:** 2014-08-28

**Authors:** Sylvia Andrzejewski, Simon-Pierre Gravel, Michael Pollak, Julie St-Pierre

**Affiliations:** 1 Goodman Cancer Research Centre, McGill University, 1160 Pine Ave. West, Montréal, QC H3A 1A3, Canada; 2 Department of Biochemistry, McGill University, 3655 Promenade Sir William Osler, Montréal, QC H3G 1Y6, Canada; 3 Lady Davis Institute for Medical Research, McGill University, 3755 Côte-Sainte-Catherine, Montréal, QC H3T 1E2, Canada; 4 Cancer Prevention Center, Sir Mortimer B. Davis-Jewish General Hospital, McGill University, 3755 Côte-Sainte-Catherine, Montréal, QC H3T 1E2, Canada; 5 Department of Oncology, McGill University, 546 Pine Ave. W., Montréal, QC H2W 1S6, Canada

**Keywords:** Metformin, Mitochondria, Respiration, Complex I, Cancer, Metabolism, Citric acid cycle

## Abstract

**Background:**

Metformin is widely used in the treatment of diabetes, and there is interest in ‘repurposing’ the drug for cancer prevention or treatment. However, the mechanism underlying the metabolic effects of metformin remains poorly understood.

**Methods:**

We performed respirometry and stable isotope tracer analyses on cells and isolated mitochondria to investigate the impact of metformin on mitochondrial functions.

**Results:**

We show that metformin decreases mitochondrial respiration, causing an increase in the fraction of mitochondrial respiration devoted to uncoupling reactions. Thus, cells treated with metformin become energetically inefficient, and display increased aerobic glycolysis and reduced glucose metabolism through the citric acid cycle. Conflicting prior studies proposed mitochondrial complex I or various cytosolic targets for metformin action, but we show that the compound limits respiration and citric acid cycle activity in isolated mitochondria, indicating that at least for these effects, the mitochondrion is the primary target. Finally, we demonstrate that cancer cells exposed to metformin display a greater compensatory increase in aerobic glycolysis than nontransformed cells, highlighting their metabolic vulnerability. Prevention of this compensatory metabolic event in cancer cells significantly impairs survival.

**Conclusions:**

Together, these results demonstrate that metformin directly acts on mitochondria to limit respiration and that the sensitivity of cells to metformin is dependent on their ability to cope with energetic stress.

## Background

The biguanide metformin is well established as an important drug in the treatment of type II diabetes [1-3]. Pharmaco-epidemiologic evidence [4,5] and laboratory models [6,7] have suggested that metformin may have antineoplastic actions, and this has led to renewed interest in the molecular actions of the drug [8]. One popular view is that metformin acts as an inhibitor of complex I of the electron transport chain. However, the notion that metformin acts directly on mitochondria to inhibit complex I is controversial [9-15]. Recent work on the sensitivity of cancer cells to the direct actions of metformin further highlighted the controversy surrounding the mode of action of metformin. These studies demonstrate that cancer cells that are deficient in mitochondrial functions (rho0 cells) are sensitive to the action of metformin [11], and that cancer cells harboring complex I mutations are more sensitive to the action of metformin compared with cancer cells without these mutations [16].

While there is controversy regarding the molecular mechanisms underlying the action of metformin, there is a general agreement that the drug causes energetic stress, and that this results in a variety of cell lineage-specific secondary effects. The liver is an important target organ in the context of diabetes. This organ is exposed to a relatively high concentration of metformin via the portal circulation following oral ingestion, and hepatocytes express high levels of membrane transporters required for drug influx [17]. Metformin-induced hepatocyte energetic stress leads to a reduction in gluconeogenesis [18-20], leading to improvements in hyperglycemia and hyperinsulinemia. These metabolic actions also represent a candidate mechanism relevant to the subset of cancers that are insulin-responsive [21]. Recent work has indicated that metformin treatment alters the hepatocellular redox state by inhibiting mitochondrial glycerophosphate dehydrogenase [22].

Understanding the actions of metformin on energy metabolism, particularly on mitochondrial functions, is important in the context of interest in ‘repurposing’ the compound for possible applications in oncology. There is increasing evidence that mitochondrial metabolism plays an important role in supporting tumor growth, by providing ATP as well as metabolic intermediates that can be used for anabolic reactions [23]. Also, functional mitochondrial complex I has been shown to be essential for the promotion of aerobic glycolysis and the Warburg effect [24]. In support of these points, PGC-1α or ERRα, two known central regulators of mitochondrial metabolism have been shown to promote the growth of liver, colon, breast, prostate and melanoma cancers [25-29]. Here, we demonstrate the influence of metformin on mitochondrial bioenergetics in cells and in isolated mitochondria.

## Methods

### Animals, cells and reagents

Wild-type male C57BL/6J mice were purchased from The Jackson laboratory (Bar Harbour, ME, USA). NT2196 and NMuMG cells were kindly provided by Dr. William Muller (McGill University, Montréal, Canada) and have been described elsewhere [30]. MCF7 and MCF10A cells were purchased from ATCC. All reagents were purchased from Sigma-Aldrich unless otherwise stated.

### Cell culture

All cell culture material was purchased from Wisent Inc. unless otherwise specified. NT2196 and NMuMG cells were grown as previously published [30]. MCF7 cells were grown in Dulbecco’s Modified Eagle Medium (DMEM) media with 10% fetal bovine serum, supplemented with penicillin and streptomycin. MCF10A cells were grown in DMEM/Ham’s F12 50/50 Mix Media supplemented with 5% horse serum, 20 ng/mL human epidermal growth factor (hEGF), 0.5 μg/mL hydrocortisone, 10 μg/mL insulin, penicillin and streptomycin. All cells were grown at 37°C, 5% CO_2_ (Thermo Forma, Series II Water Jacketed CO_2_ Incubator). For the experiments comparing the impact of growth in glucose or galactose media on respiration, MCF7 cells were cultured either in standard glucose DMEM or in galactose (25 mM) medium that has the same composition as DMEM except that the glucose has been replaced with galactose. Cells were cultured in glucose or galactose medium for a period of 20 to 25 days after being put into culture. Cells were then treated with either ddH_2_0 (control) or metformin (0.5 mM) for a period of 24 hours, after which respiration was assessed as previously described [31].

### Cell proliferation

A fixed number of cells were plated in 6-well plates (9.6 cm^2^/well). Every 24 hours, the media was removed and cells were treated with ddH_2_0 (control) or metformin (0.5 mM and 5.0 mM). At the respective time points (24, 48, and 72 hours), the media was removed and stored into tubes (to collect floating cells); the adherent cells were washed with phosphate buffered saline (PBS), trypsinized and resuspended in the media collected, which was centrifuged at 2,500 rpm for 5 minutes. The media was removed (and used for lactate and glucose measurements; The media was removed (and used for lactate and glucose fold change measurements in the presence of metformin) and the cell pellet was resuspended), and the cell pellet was resuspended in a known volume of fresh media. Both total and live cell counts were obtained using Trypan Blue Stain (0.4%, Gibco) and a TC10 automated cell counter (Bio-Rad).

### Lactate and glucose concentration

MCF10A, MCF7, NT2196 and NMuMG cells were grown in 6-well plates (9.6 cm^2^/well) to 60% confluency. The media in each well was removed and centrifuged at 13,000 rpm for 10 minutes to remove cellular debris, placed into new tubes and analyzed with a Nova BioProfile 400 analyzer. Wells that contained only media in the absence of cells were also analyzed in this manner to serve as blanks. To account for cell number, cells were counted as described above. To calculate lactate production and glucose consumption, the concentration of either lactate or glucose present in each condition was subtracted from that of blank wells and this value was then normalized for total cell count.

### Respiration

Respiration measurements with cultured cells or isolated mitochondria were performed using a Digital Model 10 Clark Electrode (Rank Brothers, Cambridge, UK). Respiration with cultured cells was carried out in their respective growth medium while respiration with isolated mitochondria was carried out in KHEB (120 mM KCl, 5 mM KH_2_PO_4_, 3 mM 4-(2-hydroxyethyl)-1-piperazineethanesulfonic acid (HEPES), 1 mM ethylene glycol tetraacetic acid (EGTA) and 0.3% bovine serum albumin (BSA) (w/v), pH 7.2) assay medium. Respiration traces for isolated mitochondria were digitized using DigitizeIt Software (Version 1.5). This software extracts values from traces using the background graph paper found on the trace as a reference. Simply, the respiration traces were imported, the axes were defined manually based on the corresponding values found on the graph paper of the trace, and data values were generated by the software and plotted using GraphPad Prism 5 Software.

### Isolation of mitochondria from skeletal muscle

Mice were sacrificed at approximately 6 months of age with approval from the McGill University Animal Care Committee. Mitochondria from skeletal muscle were isolated as previously described [32]. The integrity of mitochondrial suspensions were evaluated by quantifying respiratory control ratio (RCR) values that are obtained by dividing the rate of oxygen consumption in the presence of ADP (state 3) by that in the presence of oligomycin (state 4). Only mitochondrial suspensions displaying RCR values greater than 3 in control conditions were used.

### Treatment of cells with metformin and respiration

NT2196, NMuMG, MFC10A and MCF7 cells were grown in the presence of ddH_2_0 (control) or specific doses of metformin for 24 hours. 1 × 10^6^ cells were used for respiration measurements. Calculations of coupled and uncoupled respiration were performed according to [31]. Briefly, coupled respiration is calculated by subtracting total respiration from oligomycin-insensitive (2.5 μg/mL/1 × 10^6^ cells) respiration. Uncoupled respiration represents oligomycin-insensitive respiration. Nonmitochondrial respiration represents respiration that is insensitive to myxothiazol (10 μM). Cells displayed no detectable nonmitochondrial respiration.

### Treatment of isolated mitochondrial suspensions with metformin and respiration

For the metformin incubation experiments, mitochondria (0.6 mg/mL) were incubated in KHEB media at 37°C in a temperature-controlled water bath (Fisher Scientific, Isotemp 3006S) in the presence of either complex I (equimolar 30 mM malate and pyruvate) or complex II (25 mM succinate and 50 μM rotenone) substrates, either in the presence of ddH_2_0 (control) or 10 mM metformin for 30 minutes. Samples were resuspended every 10 minutes. After 30 minutes, the 100 μL reaction was diluted in 400 μL KHEB media (final equimolar concentration of 6 mM malate and pyruvate or 5 mM succinate and 10 μM rotenone, in the absence or presence of 2 mM metformin). Respiration was recorded immediately, followed by the addition of ADP (500 μM, state 3), oligomycin (2.5 μg oligomycin/mg mitochondrial protein, state 4) and FCCP (1.5 μM).

### Stable isotope tracer analyses in cells and isolated mitochondria

MCF10A and MCF7 cells were cultured in 6-well plates (9.6 cm^2^/well) to 80% confluency, after which ddH_2_0 (control) or metformin (0.5 mM, 5.0 mM) was added to the media for 24 hours. The media was then exchanged for [U-^13^C]glucose (Cambridge Isotope Laboratories, Tewksbury, MA, USA, CLM-1396, 99% atom ^13^C)-labeled media for a period of 1 hour. Cells were then rinsed once with 4°C saline solution (9 g/L NaCl) and quenched with 80% methanol (<20°C). Isolated mitochondria from murine skeletal muscle were resuspended in KHEB media at a concentration of 1.5 mg/mL. Samples were incubated in a temperature-controlled water bath (Fisher Scientific, Isotemp 3006S) at 37°C in the presence of 1 mM malate and 1 mM [U-^13^C]pyruvate for 30 minutes, either in the presence of ddH_2_0 (control) or 5 mM metformin. Samples were then quenched in 80% methanol (<20°C). The remaining procedure is identical for cellular and mitochondrial extracts. Metabolite extraction was carried by sonication at 4°C (10 minutes, 30 sec on, 30 sec off, high setting, Diagenode Bioruptor). Extracts were cleared by centrifugation (14,000 rpm, 4°C) and supernatants were dried in a cold trap (Labconco) overnight at -4°C. Pellets were solubilized in pyridine containing methoxyamine-HCl (10 mg/mL) by sonication and vortex, centrifuged and pellets were discarded. Samples were incubated for 30 minutes at 70°C (methoximation), and then were derivetized with MTBSTFA at 70°C for 1 h. Next, 1 μL was injected into an Agilent 5975C GC/MS configured for single ion monitoring (SIM) according to [33]. Data analyses were performed using the Chemstation software (Agilent, Santa Clara, USA). Mass isotopomer distribution analyses were performed according to [34,35].

## Results

### Cancer cells devote a larger fraction of their respiration to uncoupled reactions than nontransformed cells

**Figure 1:**
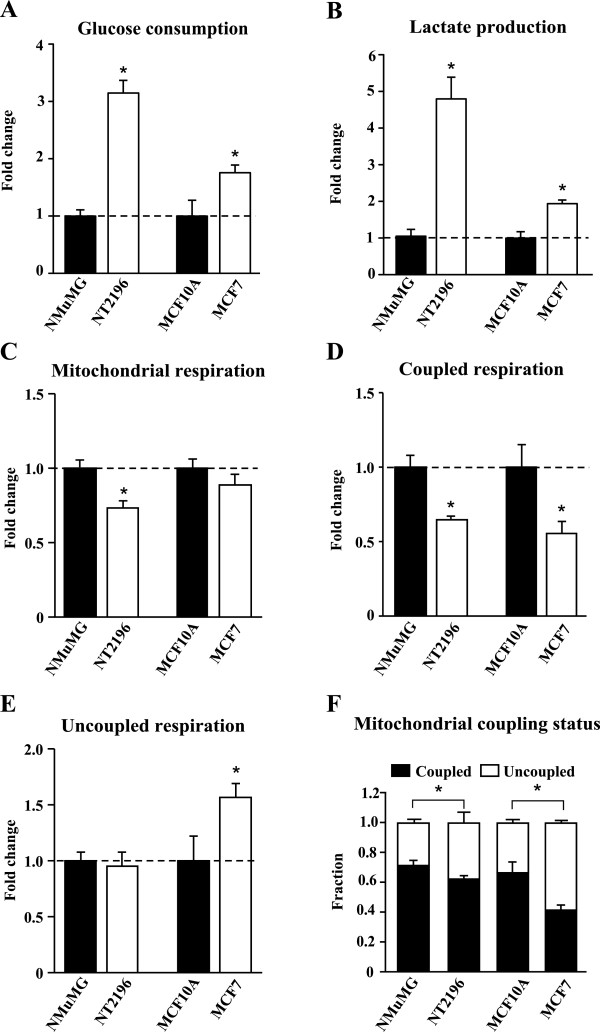
**Mitochondrial respiration in cancer cells is more uncoupled from ATP production than that in nontransformed cells.** Aerobic glycolysis and mitochondrial respiration were quantified in murine breast cancer cells (NT2196) and parental controls (NMuMG) as well as in human breast cancer cells (MCF7) and nontransformed controls (MCF10A). **(A)** Glucose consumption and **(B)** lactate production in cancer cells are presented as fold change from controls. **(C)** Total mitochondrial respiration, **(D)** coupled respiration and **(E)** uncoupled respiration in cancer cells are presented as fold change from controls. **(F)** The fraction of mitochondrial respiration devoted to coupled and uncoupled respiration was calculated by dividing the rate of coupled or uncoupled respiration by that of total mitochondrial respiration. Coupled respiration is the respiration used to drive ATP synthesis. Uncoupled respiration is used to drive proton leak reactions. Data are presented as means ± SEM. n = 3. *P <0.05, Students *t-*test, where * represents a significant change from nontransformed controls.

In order to assess the dependence of breast cancer cells on aerobic glycolysis and mitochondrial respiration for ATP production, we compared these parameters in murine and human breast cancer cell lines to nontransformed controls. We used NT2196 cells that express oncogenic Neu/ErbB2 and their parental NMuMG cells as murine cell line models. For the human cell models, we used MCF7 cancer cells and MCF10A epithelial cells as comparative controls. Both NT2196 and MCF7 cancer cells displayed higher glucose consumption (Figure 1A) and lactate production (Figure 1B) compared to their respective controls. These data confirm that aerobic glycolysis is elevated in cancer cells compared with nontransformed cells. We next assessed mitochondrial respiration in breast cancer cells and nontransformed controls. Mitochondrial respiration can be coupled (linked to ATP production), or uncoupled (driving proton leak reactions). NT2196 cancer cells showed reduced mitochondrial respiration compared with NMuMG control cells (Figure 1C). The reduced mitochondrial respiration was due to a decrease in coupled respiration (Figure 1D). Uncoupled respiration was similar between murine cancer cells (NT2196) and their parental controls, while it was increased in human breast cancer cells (MCF7) compared to control cells (Figure 1E). Coupled respiration (Figure 1D) was also decreased in MCF7 cells compared with controls, leading to no significant change in overall mitochondrial respiration compared with controls (Figure 1C). Next, we quantified the mitochondrial coupling status by calculating the fraction of mitochondrial respiration that was coupled and uncoupled to ATP production. Breast cancer cells devoted a larger fraction of their mitochondrial respiration to drive uncoupling reactions compared with controls (Figure 1F). Conversely, control cells devoted a larger fraction of their mitochondrial respiration to support ATP production (Figure 1F). Together, these data demonstrate that these breast cancer cells have higher aerobic glycolysis rate than controls, and that their mitochondria favor uncoupling reactions.

### Metformin causes a dose-dependent increase in the proportion of uncoupled respiration

**Figure 2:**
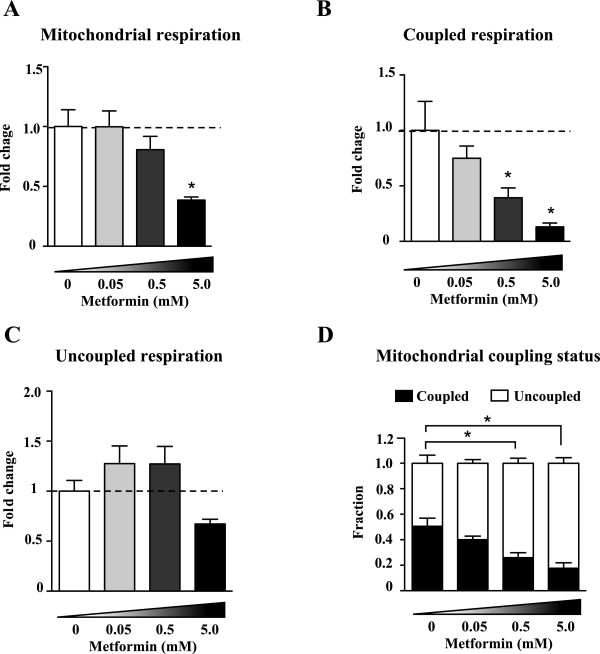
**Dose-dependent effects of metformin on mitochondrial respiration. (A)** Total, **(B)** coupled and **(C)** uncoupled respiration in MCF7 cells after 24 hours of treatment with ddH_2_O (control) or metformin of varying concentrations (0.05, 0.5 and 5.0 mM). Fold change represents the change in respiration from untreated samples. **(D)** The fraction of mitochondrial respiration devoted to coupled and uncoupled respiration was calculated as in Figure 1. Data are presented as means ± SEM. n = 4 to 5. *P <0.05, One-way ANOVA followed by a Dunnet’s multiple comparison test.

Metformin caused a dose-dependent decrease in respiration in MCF7 cancer cells (Figure 2A). This decrease in respiration was due to a reduction in the rate of respiration used for ATP synthesis (Figure 2B). The rate of uncoupled respiration was unaffected by the lower doses of metformin, but decreased at 5 mM (Figure 2C). The fact that the rate of coupled respiration decreased with increasing doses of metformin, while uncoupled respiration remained mostly unaffected, caused breast cancer cells to devote an increasingly higher proportion of their respiration for uncoupled reactions (Figure 2D). Together, these results demonstrate that metformin decreases mitochondrial respiration, and has a profound impact on the ability of mitochondria to generate ATP.

### Metformin leads to a greater upregulation of aerobic glycolysis in cancer cells than nontransformed controls

**Figure 3:**
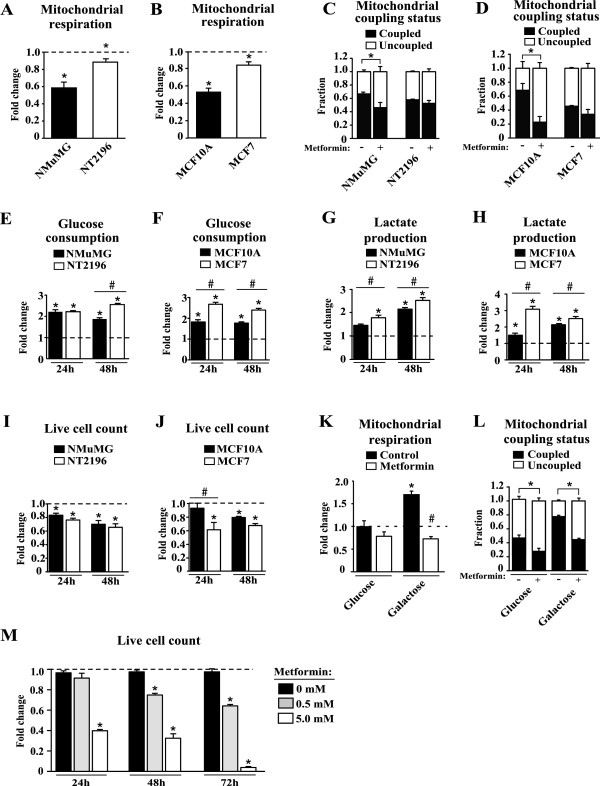
**Sensitivity of cells to metformin is dependent on the capacity to engage in aerobic glycolysis. (A-B)** Total respiration is presented as fold change upon metformin treatment (0.5mM) from untreated conditions. **(C-D)** The mitochondrial coupling status represents coupled and uncoupled respiration as a fraction of total mitochondrial respiration, for both untreated and treated conditions. **(E-F)** Glucose consumption, **(G-H)** lactate production and **(I-J)** live cell counts of cells treated with metformin (5 mM) for either 24 or 48 hours are represented as a fold change from untreated conditions. **(K)** Mitochondrial respiration of MCF7 cells grown in glucose or galactose media in the presence of ddH_2_0 (control) or metformin (0.5 mM) for 24 hours. Data are normalized to the respiration rate of MCF7 cells in the presence of glucose without metformin. **(L)** The fractions of mitochondrial respiration devoted to coupled and uncoupled respiration were calculated as in C-D. **(M)** Live cell counts for MCF7 cells cultured in galactose media with treatment of metformin (0.5 or 5.0 mM) for periods of 24, 48 and 72 hours, are represented as a fold change from untreated conditions. For **(A-D)**, Data are presented as means ± SEM. n = 4, where *P <0.05, Student’s *t-*test. For **(E-J,L,M)**, data are presented as means ± SEM. n = 3, # and *P <0.05, Student’s *t-*test, where * represents a significant change from untreated conditions and # represents a significant change between indicated cell lines. For **(K)**, data are presented as means ± SEM. n = 3, # and *P <0.05, Student’s *t-*test, where * represents a significant change from the respiration rate of MCF7 cells in the presence of glucose without metformin, while # represents a significant change from the respiration rate of MCF7 cells in the presence of galactose without metformin.

As metformin had a significant impact on mitochondrial metabolism in breast cancer cells (Figure 2), we then compared the effect of this drug between cancer cells and nontransformed controls given that they display differences in mitochondrial metabolism (Figure 1). Metformin caused a decrease in mitochondrial respiration in both breast cancer cells and nontransformed controls (Figures 3A,B). However, the decrease in respiration was larger in nontransformed cells compared with breast cancer cells (Figure 3A,B). Metformin also caused a decrease in respiration upon acute treatment (15 minute incubation), [see Additional file 1, Additional file 2: Figure S1] in the murine control cells (NMuMG), while no change was observed in the murine breast cancer cells (NT2196). Furthermore, metformin caused a shift in the mitochondrial coupling status in favor of uncoupled respiration, which was greater in magnitude in nontransformed cells compared with cancer cells (Figure 3C,D).Both cancer cells and nontransformed controls displayed elevated aerobic glycolysis upon metformin exposure (Figures 3E-H). This upregulation of glycolysis will mitigate the drop in ATP production by mitochondria caused by metformin. Cancer cells elicited significantly larger increases in aerobic glycolysis in the presence of metformin than controls (Figures 3E-H). Despite the greater compensatory increase in aerobic glycolysis by cancer cells, their proliferation was equally or even more affected by metformin treatment than controls (Figures 3I,J). Indeed, the proliferation of NMuMG and NT2196 was affected similarly by metformin treatment (Figure 3I), while that of MCF7 was more affected than MCF10A at earlier time points (Figure 3J). However, all cell lines showed reduced cell proliferation in the presence of metformin compared to untreated conditions (Figure 3 I,J). Overall, failure of the greater compensatory increase in glycolysis by cancer cells to confer a survival advantage in the presence of metformin illustrates that these cells are more energetically stressed by metformin than nontransformed controls, consistent with the view that transformation is associated with increased ATP demand.

An important implication of these data is that a constant supply of glucose to cells is critical to attenuate the energetic stress caused by metformin by fuelling aerobic glycolysis. Therefore, we tested whether cells that are forced to rely exclusively on mitochondrial metabolism for ATP production are more sensitive to metformin. We cultured human breast cancer cells (MCF7) in media where the glucose had been replaced by galactose [36]. MCF7 cells grown in galactose media displayed an approximate two-fold increase in mitochondrial respiration compared with MCF7 cells grown in glucose media (Figure 3K). Importantly, MCF7 cells grown in galactose media devoted a larger proportion of their respiration for ATP production than those grown in glucose (Figure 3L). These results validate the experimental design by showing that cancer cells grown in the presence of galactose increase mitochondrial respiration, and elevate the proportion of their mitochondrial respiration devoted to support ATP production compared to cells grown in glucose (Figure 3K,L). Metformin caused an approximate 20% decrease in respiration for MCF7 cells grown in glucose media (Figure 3K). However, when MCF7 cells were grown in galactose media, metformin had a more profound impact on mitochondrial respiration, which decreased by more than two-fold upon metformin treatment (Figure 3K). Metformin caused a significant increase in the proportion of uncoupled respiration for MCF7 cells grown in either glucose or galactose (Figure 3L). However, the impact of metformin on the proportion of uncoupled respiration was much greater for MCF7 cells grown in galactose than glucose, given that at baseline, these cells were more coupled than those grown in glucose (Figure 3L). Importantly, MCF7 cells grown in galactose media and exposed to 5 mM metformin for 48 hours exhibited strikingly more cell death than MCF7 cells grown in glucose media (Figure 3J,M). Together, these results demonstrate that cells that cannot engage aerobic glycolysis due to limiting glucose levels, are entirely dependent on mitochondria for ATP production, and are thus more susceptible to the action of metformin.

### Metformin diminishes glucose metabolism through the citric acid cycle

**Figure 4:**
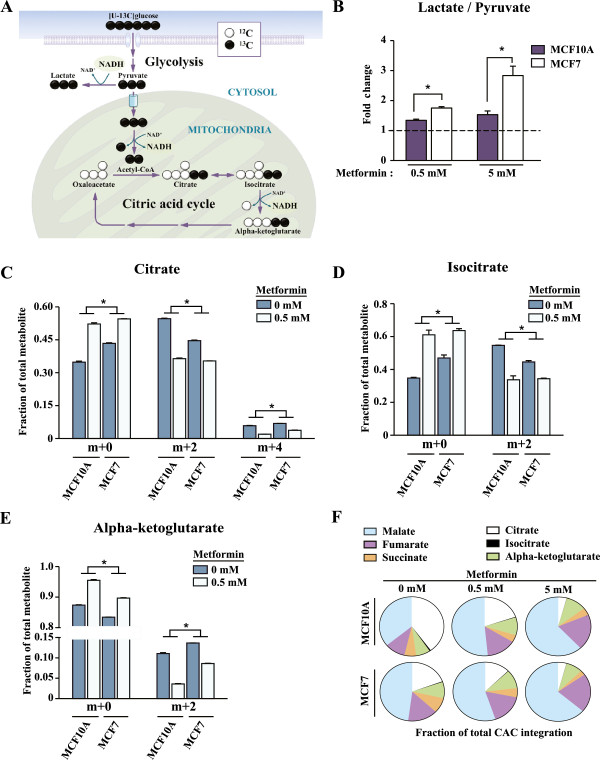
**Metformin reduces glucose metabolism through the citric acid cycle. (A)** Schematic depicting glucose carbon flow into glycolysis and the citric acid cycle (CAC). The usage of tracer metabolites such as [U-^13^C]glucose where all carbons (^12^C, white) are replaced by ^13^C (black circles) allows for the measurement of CAC activity by gas chromatography/mass spectrometry (GC/MS) analysis and isotopomer enrichments. **(B-F)** MCF7 and MCF10A cells were treated with ddH_2_O (control) or metformin (0.5 mM or 5.0 mM) for 24 hours. Cells were then incubated with [U-^13^C]glucose (m + 6) for 1 hour. **(B)** Intracellular lactate to pyruvate ratio induced by metformin treatment, displayed as fold change from untreated conditions. **(C)** Enrichment of citrate (m + 2) and (m + 4), **(D)**, isocitrate (m + 2) **(E)** and alpha-ketoglutarate (m + 2) upon incubation with [U-^13^C]glucose and quantified as mass isotopomer distributions. **(F)** CAC intermediates reorganization upon metformin treatment. The sum of the ion intensities for all the isotopomers of each individual CAC intermediate was normalized to the sum of the ion intensities for all the isotopomers of all combined CAC intermediates. For **B-E**, data are presented as mean ± SEM of a representative experiment performed in triplicate of three independent experiments for control and 0.5 mM metformin treatments, and two independent experiments for 5.0 mM metformin treatment. *P <0.05, Student’s *t*-test. For **F**, data are presented as mean of a representative experiment performed in triplicate of three independent experiments for control and 0.5 mM metformin treatments, and two independent experiments for 5.0 mM metformin treatment. CAC: citric acid cycle.

Metformin caused a decrease in mitochondrial respiration in breast cancer cells as well as in nontransformed controls (Figures 2 and 3). Given the intimate link between the activity of the electron transport chain and the citric acid cycle [33,37,38], we investigated the impact of metformin on glucose metabolism through the citric acid cycle in MCF10A and MCF7 cells. In order to address this question, we performed stable isotope tracer analyses using [U-^13^C]glucose labeled on all six carbons (m + 6). Glucose (m + 6) will generate pyruvate (m + 3) through glycolysis (Figure 4A). Pyruvate (m + 3) can then be converted into lactate (m + 3) through aerobic glycolysis or into citric acid cycle intermediates (m + 2) through mitochondrial metabolism (Figure 4A). Metformin decreased the labeling of citrate, isocitrate and alpha-ketoglutarate (m + 2) through the citric acid cycle in MCF7 cancer cells and controls (Figures 4C-E). These data indicate that less glucose is entering mitochondrial metabolism in cells treated with metformin compared with untreated cells. Furthermore, the proportion taken by citric acid cycle intermediates within the citric acid cycle changed considerably upon metformin exposure (Figure 4F). Indeed, cells treated with metformin exhibited a reduced fraction of citrate and an increased fraction of malate within the citric acid cycle (Figure 4F). MCF10A cells displayed more drastic citric acid cycle rearrangement upon metformin treatment than MCF7 cells, supporting the notion that cells with high mitochondrial metabolism are more metabolically responsive to metformin. Contrary to the decrease in the metabolism of glucose in mitochondria, metformin caused an increase in the intracellular lactate to pyruvate ratio in cancer cells and controls, illustrating that metformin stimulates aerobic glycolysis (Figure 4B). This result is in agreement with the data presented in Figure 3 using a different technology. Globally, these experiments demonstrate that cancer cells treated with metformin increase the activity of glycolysis, while decreasing that of the mitochondrial citric acid cycle.

### Metformin decreases respiration in isolated mitochondria

**Figure 5:**
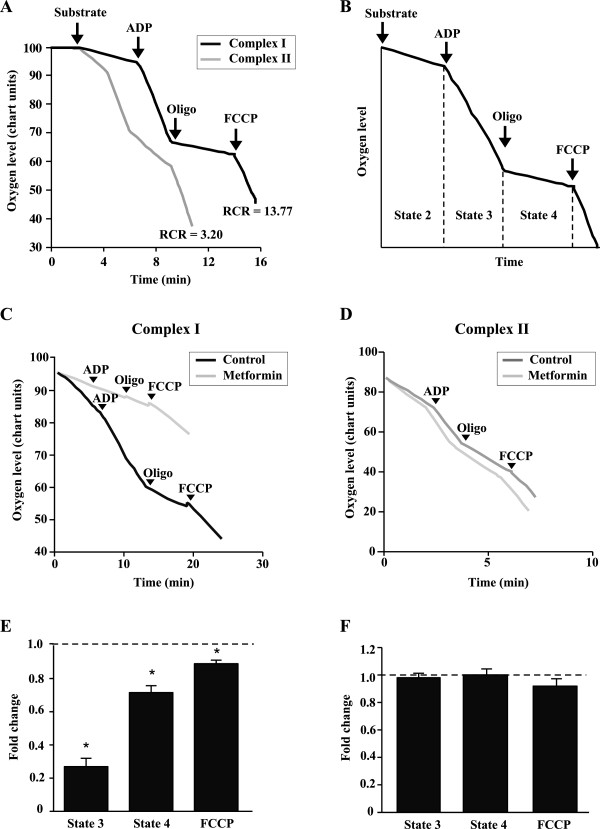
**Metformin directly acts on mitochondria to inhibit respiration. (A-B)** Design of experiments with isolated mitochondria from murine skeletal muscle. Mitochondria were incubated with either complex I (malate and pyruvate) or complex II (succinate and rotenone) substrates. Typical respiratory control ratio (RCR) values are shown for mitochondria respiring on either complex I or II substrates. Respiration in the presence of substrates is called state 2. Respiration in the presence of ADP where mitochondria are using ADP to make ATP is called state 3. Respiration in the presence of oligomycin where mitochondria are driving proton leak reactions is called state 4. FCCP stimulates uncoupled respiration and represents the maximal respiratory capacity. RCR values are calculated by dividing the rate of respiration in state 3 by that in state 4 and are indicative of the integrity of the mitochondrial suspensions. **(C-F)** Mitochondria isolated from murine skeletal muscle were incubated with complex I **(C,E)** or complex II **(D,F)** substrates and treated with ddH_2_O (control) or metformin (2 mM) **(E-F)**. Respiration rates are expressed as the fold difference from untreated mitochondria. Data are presented as means ± SEM. n = 3. *P <0.05, Student’s *t-*test.

Metformin has a profound impact on mitochondrial metabolism in cells (Figures 2, 3 and 4). In order to assess whether metformin can directly act on mitochondria, we tested the impact of metformin on the respiration of isolated mitochondrial suspensions using mitochondria isolated from skeletal muscle of mice (Figure 5) or from MCF10A and MCF7 cells [see Additional file 1, Additional file 2: Figures S2 and S3]. The quality of mitochondrial suspensions was evaluated using RCR values that are obtained by dividing the rate of oxygen consumption when mitochondria are actively synthesizing ATP (state 3), by that when they are driving proton leak reactions (state 4) [39]. The quality of mitochondrial suspensions isolated from murine skeletal muscle was high, with RCR values above 10 (Figures 5A,B).

To probe the impact of metformin on mitochondria, we used mitochondria that were incubated with either complex I or II substrates. Comparison of the effect of metformin on the respiration rate of mitochondria that were incubated with complex I or II substrates allows one to pinpoint whether metformin is acting on complex I or II, given that complexes III to V are involved in both complex I- and II-dependent respiration. Metformin reduced state 3 and state 4 respiration, as well as the maximal respiratory capacity of mitochondria respiring on complex I substrates (Figure 5C,E), but had no significant effect on these parameters when mitochondria were respiring on complex II substrates (Figure 5D,F). Finally, metformin also acutely decreased complex I-dependent respiration in isolated mitochondria from cultured MCF7 and MCF10A cells [see Additional file 1, Additional file 2: Figures S2 and S3]. Together, these results demonstrate that metformin can directly act on mitochondria and limit complex I-dependent respiration.

### Metformin reduces citric acid cycle activity in isolated mitochondria

**Figure 6:**
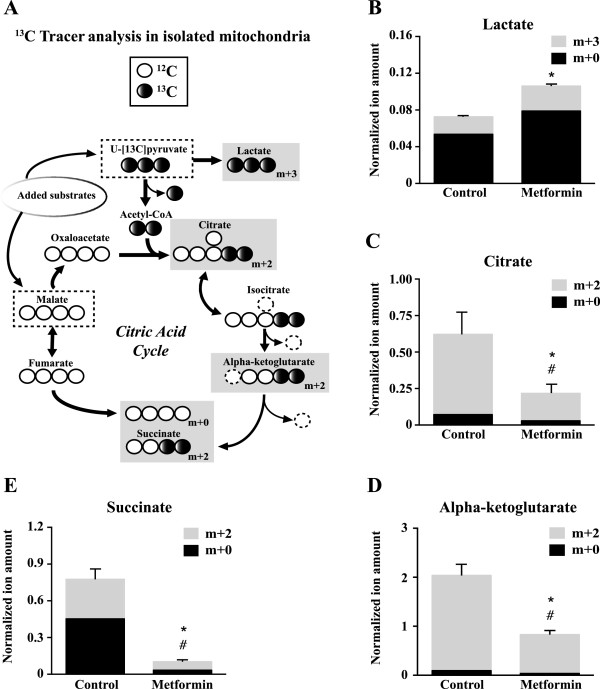
**Metformin inhibits citric acid cycle activity in isolated mitochondria.** Mitochondria were incubated with [U-^13^C]pyruvate (m + 3) and unlabeled malate in the presence of ddH_2_O (control) or metformin (5 mM) for 30 minutes. **(A)** Schematic depicting stable isotope tracer experiment where substrates used are uniformly labeled [U-^13^C]pyruvate and unlabeled malate. The metabolites analyzed in B-E are placed into gray boxes where the isotopic enrichment is written as *m + k* where *k* is the number of ^13^C (black circles). **(B)** Enrichment of lactate (m + 3), **(C)**, citrate (m + 2), **(D)** alpha-ketoglutarate (m + 2) and **(E)** succinate (m + 2) as evaluated by GC/MS analysis of mass distributions. Data are expressed as normalized ion amount which represents values obtained from mass isotopomer distribution (MID) × corrected area. Data are presented as means ± SEM. n = 3. *P <0.05, Student’s *t*-test (m + 2 or m + 3). #*P* <0.05, Student’s *t*-test (m + 0).

Given that metformin can directly inhibit complex I-dependent respiration in isolated mitochondria, we assessed whether metformin could impact the metabolism of substrates through the citric acid cycle as observed in intact cells (Figure 4). In order to do this, we performed stable isotope tracer experiments in isolated mitochondria [34]. Mitochondria were incubated with labeled $\left[U^{-13},C\right]$pyruvate (m + 3) and unlabeled malate (Figure 6A). The $\left[U^{-13},C\right]$pyruvate (m + 3) generates m + 2 citric acid cycle intermediates (Figure 6A). The $\left[U^{-13},C\right]$pyruvate (m + 3) can also generate lactate (m + 3) given that the enzyme lactate dehydrogenase is associated with skeletal muscle mitochondria [40]. Metformin decreased the generation of m + 2 citrate, alpha-ketoglutarate and succinate (Figures 6C-E), illustrating reduced metabolism of pyruvate through the citric acid cycle. The reduced usage of pyruvate through the citric acid cycle during metformin treatment was accompanied by an increase in the generation of lactate (m + 3; Figure 6B), demonstrating that pyruvate is diverted away from mitochondrial metabolism. Therefore, the reduced metabolism of pyruvate through the citric acid cycle in intact cells upon metformin treatment (Figure 4) can be captured in isolated mitochondria (Figure 6).

## Discussion

**Figure 7:**
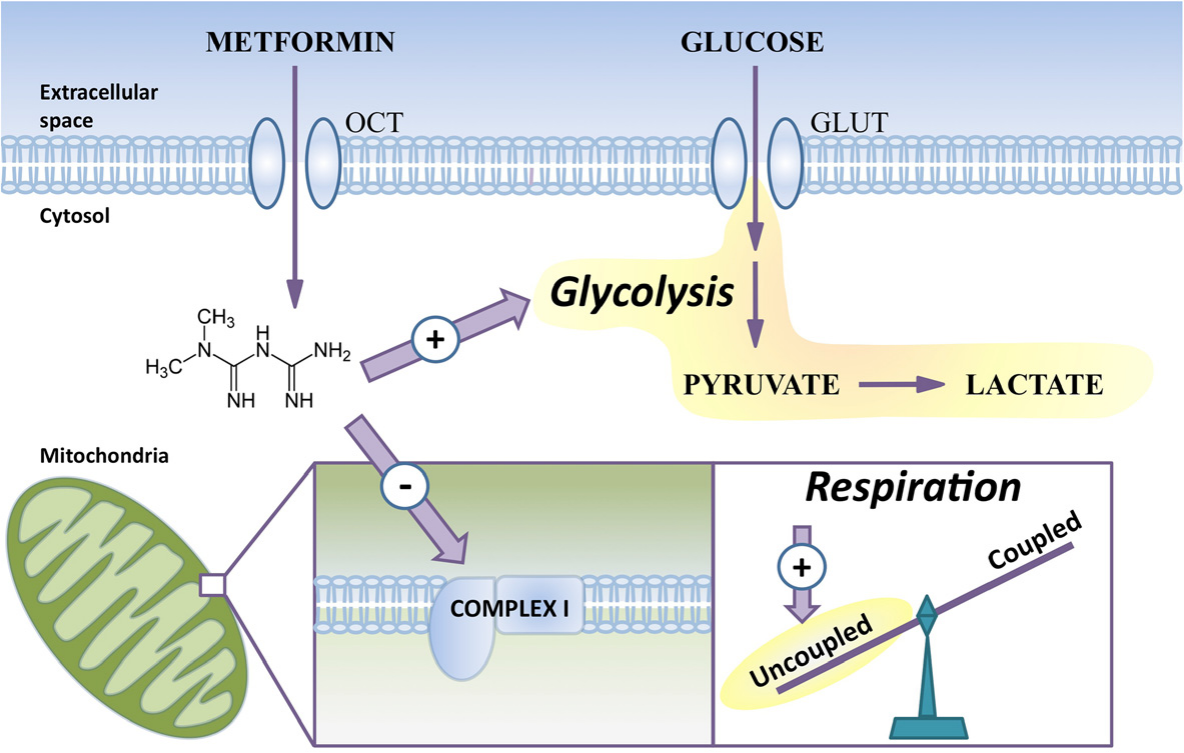
**Metformin directly acts on mitochondria and shifts the balance between coupling and uncoupling reactions.** Metformin is transported into cells through the OCT family of transporters, where it acts on mitochondria to inhibit complex I-dependent respiration and increase the proportion of uncoupled respiration. Cells respond by increasing glycolysis, ultimately leading to increased lactate production.

Although metformin is widely used in the treatment of type II diabetes, and is under investigation for possible utility in cancer treatment, its effects on cellular and mitochondrial metabolism are incompletely understood. We show that metformin acts directly on mitochondria to inhibit complex I-mediated mitochondrial respiration and citric acid cycle functions. In agreement with our results obtained with isolated mitochondria, cells treated with metformin display reduced glucose metabolism through the citric acid cycle, in addition to showing an overall decrease in mitochondrial respiration, and a shift in favor of uncoupling reactions. As a result, mitochondrial metabolism becomes energetically inefficient, and cells compensate for this limitation in ATP production by increasing aerobic glycolysis (Figure 7).

Our results confirm that mitochondria are key targets of metformin despite reports suggesting cytoplasmic actions [11,13]. This is in keeping with prior evidence for an inhibitory effect on complex I together with a membrane potential-driven accumulation of positively charged drug within the mitochondrial matrix [14]. Our data argue against an indirect action of metformin on mitochondria [9]. While this manuscript was in preparation, a study by the Chandel group has shown that the ability of metformin to limit tumour growth *in vivo* is dependent on mitochondrial complex I [41]. Also, a study by the Hirst group has demonstrated that metformin can limit the activity of purified complex I [42]. These papers support our data showing a direct effect of metformin on mitochondrial respiration.

There is clinical [43] and experimental [44] evidence that metformin use is associated with modest weight loss, in contrast to many antidiabetic medications. This is consistent with our observation that metformin causes inefficient mitochondrial metabolism, as demonstrated by the increase in the fraction of uncoupled respiration. Classic uncouplers also cause inefficient mitochondrial metabolism and have been shown to cause substantial weight loss, but are too toxic for clinical use [45]. Interestingly, recent preclinical work suggests that targeting the uncoupling agent DNP to the liver, the organ most impacted by metformin due to its pharmacokinetics following oral administration, reduces toxicity [46]. However, it is important to recognize that although metformin causes inefficient mitochondrial metabolism, it should not be considered as a classic uncoupler.

Recently, it has been shown that cancer cells that are more sensitive to low glucose are defective in oxidative phosphorylation (OXPHOS) regulation and more sensitive to biguanides [16]. The low glucose condition is a setting that is advantageous for cells displaying robust mitochondrial capacities, due to the fact that cells need to rely on alternate fuel sources that are metabolized by mitochondria [38,47]. Furthermore, because they inhibit mitochondrial metabolism, biguanides exacerbate the OXPHOS defects of cells sensitive to low glucose, explaining their greater sensitivity to metformin under low glucose conditions [16]. We found that cells cultured in the absence of glucose and in the presence of galactose displayed increased mitochondrial metabolism and were drastically more sensitive to the effects of metformin than cells grown in the presence of glucose. It has also been shown that cancer cells grown in the absence of glucose and presence of glutamine were more affected by metformin treatment than cells grown in the presence of glucose [48]. Together, these data support the notion that metformin inhibits OXPHOS, and thus cells that are forced to rely on OXPHOS are more affected by the actions of metformin. Furthermore, these data show that in the setting of inhibition of OXPHOS, cancer cells compensate by increasing glycolysis. We demonstrate that when metformin inhibits OXPHOS, either in isolated mitochondria or in intact cells, the citric acid cycle is inhibited, and accepts less glucose carbon, thus favoring lactic acid production. Importantly, if this compensation is restricted by a lack of glucose, or by inhibition of oncogenes that drive glycolysis [29,49], even in the presence of other nutrients that require mitochondrial function for generation of ATP, cell viability is threatened.

While the concept of inducing energetic stress in cancers by using metformin is appealing, pharmacokinetic issues must be considered. It is by no means clear that conventional anti-diabetic doses of metformin achieve active concentrations in neoplastic tissue. Many cancers express cell surface transport molecules such as OCT1, which are required for cellular uptake at low ambient drug concentrations, at far lower levels than in the liver, where the drug is active. However, once inside cells, the greater membrane potential of mitochondria from cancer cells [50,51] should facilitate metformin uptake compared with mitochondria from nontransformed cells. Thus, although metformin at high doses has some *in vivo* antineoplastic activity [8], it may be considered a ‘lead compound’ for pharmacokinetic optimization for possible applications in oncology.

## Conclusions

We demonstrate that metformin directly acts on mitochondria to limit citric acid cycle activity and OXPHOS, as demonstrated in isolated mitochondria as well as in intact cells. The metformin-mediated decrease in mitochondrial function was accompanied by a compensatory increase in glycolysis. Hence, the sensitivity of cells to metformin is dependent on their capacity to engage aerobic glycolysis. Biguanides could thus potentially be used in oncology to exploit the metabolic vulnerability of cancer cells.

## Abbreviations

BSA: Bovine serum albumin; CAC: Citric acid cycle; DMEM: Dulbecco’s Modified Eagle Medium; EGTA: Ethylene glycol tetraacetic acid; GC/MS: Gas chromatography/mass spectrometry; HEPES: 4-(2-hydroxyethyl)-1-piperazineethanesulfonic acid; hEGF: human epidermal growth factor; MID: Mass isotopomer distribution; OCT: Organic cation transporter; OXPHOS: Oxidative phosphorylation; PBS: Phosphate buffered saline; RCR: Respiratory control ratio; SIM: Single ion monitoring.

## Competing interests

The authors declare that they have no competing interests.

## Authors’ contribution

SA, SPG, MP and JSP planned the experiments. SA carried out mitochondrial isolations, respiration experiments and cell culture experiments. SPG carried out stable isotope tracer analyses in cells and isolated mitochondria, and SA, SPG and JSP analyzed the data. All authors contributed to the interpretation of data and writing, and all read and approved the final manuscript.

## Supplementary Material

Additional file 1 **Supplemental experimental procedures.** Acute treatment of cultured cells with metformin and respiration. Isolation of mitochondria from cultured cells. Incubation of isolated mitochondria and monitored respiration. Click here for file

Additional file 2: Figure S1 Is related to Figure 3A and Figure 3B; Metformin acutely decreases cellular respiration. Cells in suspension were treated with either ddH_2_O (control) or metformin (5 mM) for 15 minutes in a 37°C CO_2_ chamber. (A) Mitochondrial, (B) coupled, and (C) uncoupled respiration was tested immediately. N = 3, *P <0.05, Student’s *t*-test. Oxygen consumption rate represents the change in oxygen consumption (chart units) normalized per minute per 1 × 10^6^ cells, where 1 chart unit is 0.2% oxygen. **Figure S2.** is related to Figure 4; Metformin decreases respiration in isolated mitochondria from cultured cells. (A-B) Isolated mitochondria from MCF10A and MCF7 cells were incubated with either ddH_2_O(control) or metformin (10 mM) for 30 minutes in a 37°C water bath in the presence of complex I substrates (malate and pyruvate). Respiration was tested immediately (State 2), followed by the addition of ADP (State 3) and oligomycin (State 4), as indicated by the arrows above the trace. (C-D) Fold change values represent fold change from incubated but untreated mitochondria. N = 3, *P <0.05, Student’s *t*-test. **Figure S3.** is related to Figure 4; Metformin decreases respiration in a time-dependent manner in isolated mitochondria. Isolated mitochondria from murine skeletal muscle were incubated in a 37°C respiration chamber in the presence of complex I substrates: malate, pyruvate as well as oligomycin, (state 4) with the addition of either ddH_2_O (control) or metformin (10 mM) and recorded immediately at t = 0 (A). (B) The changes in respiration at the end of the recording period (t = 20 min) are represented as fold change values. Experiments were repeated for state 2 conditions (malate and pyruvate) (C) and fold change values (D) were calculated from incubated but untreated mitochondria. N = 3, *P <0.05, Student’s *t*-test. Click here for file
