# Supplementary material for: Metformin directly acts on mitochondria to alter cellular bioenergetics
Source: Cancer Metab. 2014 Aug 28;2:12. doi: 10.1186/2049-3002-2-12 (PMC4147388; doi:10.1186/2049-3002-2-12)
Supplement: Additional file 1 — Supplemental experimental procedures. Acute treatment of cultured cells with metformin and respiration. Isolation of mitochondria from cultured cells. Incubation of isolated mitochondria and monitored respiration. [file 2049-3002-2-12-S1.pdf]

SUPPLEMENTAL INFORMATION

Metformin directly acts on mitochondria to alter cellular bioenergetics

Sylvia Andrzejewski [1,2], Simon-Pierre Gravel [1,2], Michael Pollak [3,4,5], and Julie St-Pierre [1,2†]

Supplemental Experimental Procedures

Acute treatment of cultured cells with metformin and respiration

For acute treatment with metformin, untreated NT2196 or NMuMG cells were washed in PBS trypsonized and counted as previously described. $1x10^6$ cells were incubated in a volume of 100 μL of respective culture media in a 5% CO_2 incubator at 37°C for a period of 15 minutes. Cells were resuspended every 5 minutes. After 15 minutes respiration was tested as previously mentioned with oligomycin (2.5 μg/mL/$1X10^6$ cells) and myxothiazol (10 μM)

Isolation of mitochondria from cultured cells

Mitochondria were isolated as previously described (Gravel S.P *et al*. 2014) with small modifications. Briefly, cells were cultured, scraped and harvested by centrifugation, resuspended in Buffer A (250 mM sucrose, 10 mM KCl, 1 mM EDTA, 1mM EGTA, 1.5 mM $MgCl_2$, 20 mM HEPES, 1% BSA (w/v), pH 7.4) and homogenized with a Wheaton glass pestle to 80% cell lysis. The suspension was centrifuged twice at 1,000g for 7 min and once at 10,000g for 15 min. The pellet was then dissolved in KHEB buffer (120 mM

KCl, 5 mM KH_2PO_4, 3 mM HEPES, 1 mM EGTA and 0.3% BSA (w/v), pH 7.2) and mitochondrial proteins were quantified using a standard Bradford Assay with 1:3 Bradford dye reagent (BioRad).

Incubation of isolated mitochondria and monitored respiration

For metformin experiments where respiration was monitored over a period of time, mitochondria were isolated from skeletal muscle as previously described, and added to the Clarke Electrode chamber (37°C) at a concentration of 0.6 mg/mL in KHEB Buffer. To determine state 2 respiration rates, 3 mM malate and 3mM pyruvate was added at t=0 min directly to the chamber, along with either ddH_20 (control), 5 mM or 10 mM metformin. To determine state 4 respiration rates 3 mM malate, 3 mM pyruvate and 2.5 μg/mL oligomycin was added at t=0 directly to the chamber, along with either ddH_20 (control) or 5 mM metformin. Respiration was then recorded for a period of 15-20 minutes. Respiration rates during the last two minutes of recording were then determined by DigitizeIt Software and GraphPad Prism. These values were then used to determine the fold change from control.
