# Supplementary material for: Metformin directly acts on mitochondria to alter cellular bioenergetics
Source: Cancer Metab. 2014 Aug 28;2:12. doi: 10.1186/2049-3002-2-12 (PMC4147388; doi:10.1186/2049-3002-2-12)

Figure S1: is related to Figure 3A and Figure 3B ; Metformin acutely decreases cellular respiration

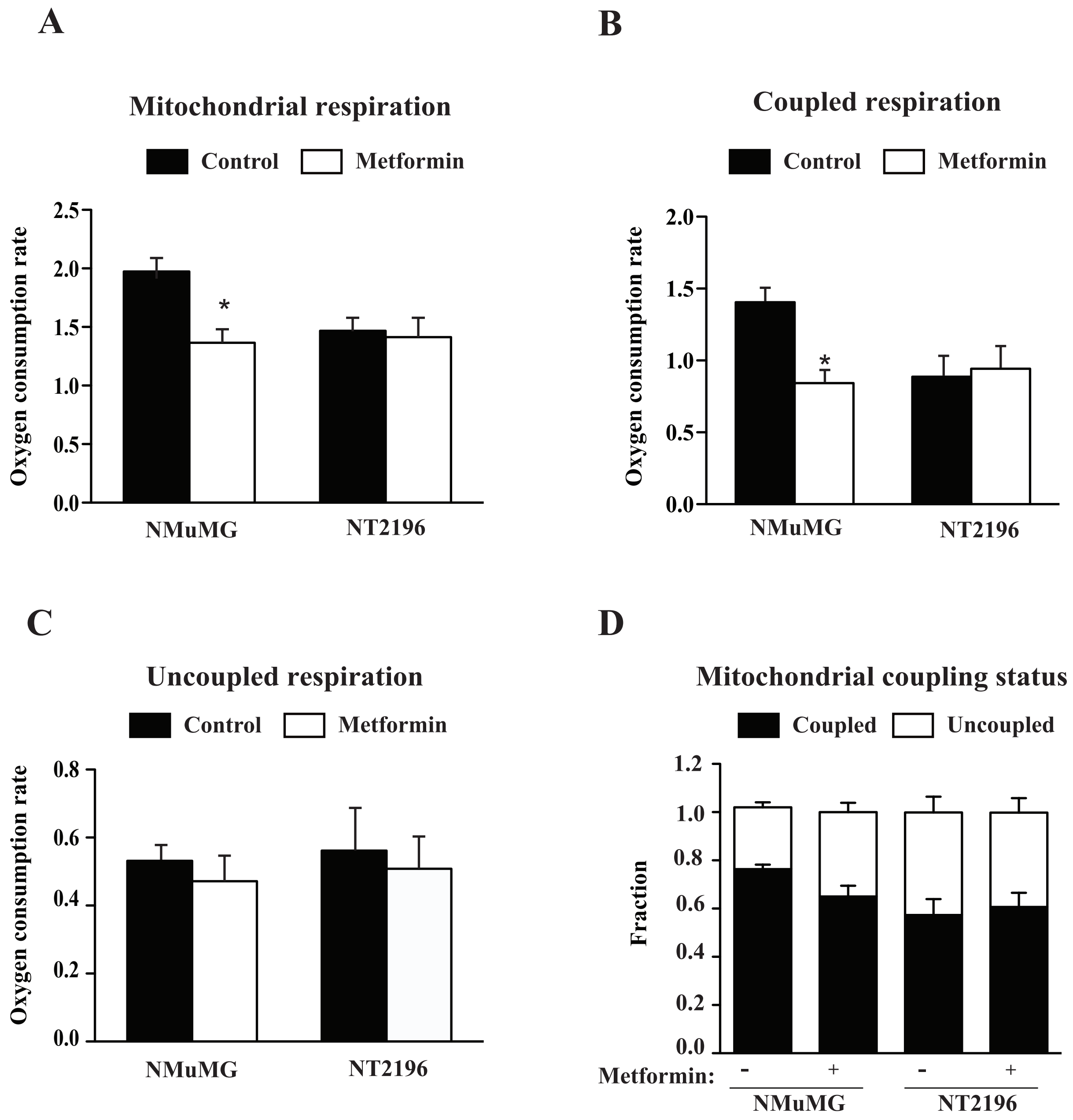

Figure S2: is related to Figure 4 ; Metformin decreases respiration in isolated mitochondria from cultured cells

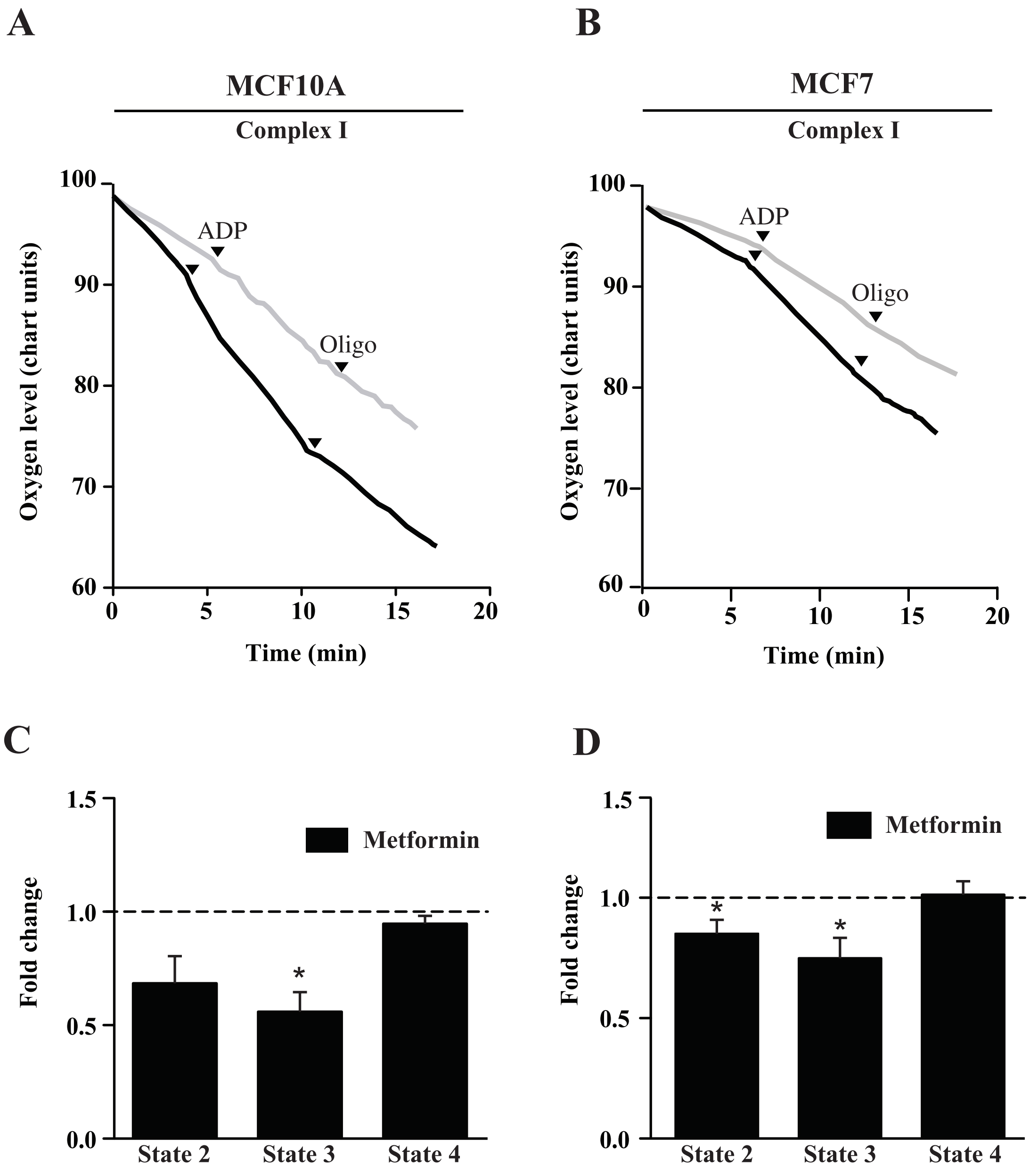

Figure S3: is related to Figure 4; Metformin decreases respiration in a time-dependent manner in isolated mitochondria

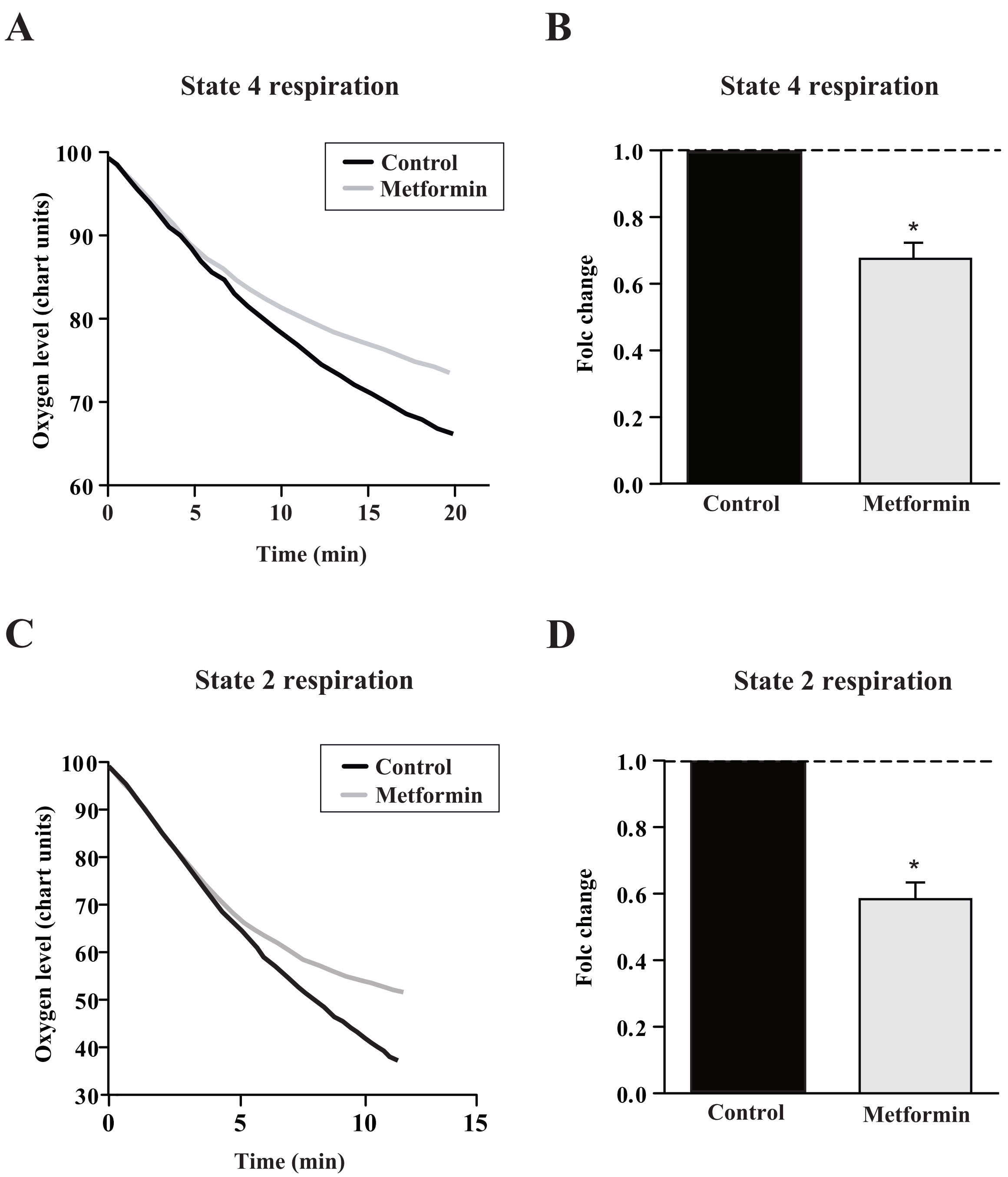

Supplement: Additional file 2: Figure S1 — Is related to Figure 3A and Figure 3B; Metformin acutely decreases cellular respiration. Cells in suspension were treated with either ddH2O (control) or metformin (5 mM) for 15 minutes in a 37°C CO2 chamber. (A) Mitochondrial, (B) coupled, and (C) uncoupled respiration was tested immediately. N = 3, *P <0.05, Student’s t-test. Oxygen consumption rate represents the change in oxygen consumption (chart units) normalized per minute per 1 × 10^6^ cells, where 1 chart unit is 0.2% oxygen. Figure S2. is related to Figure 4; Metformin decreases respiration in isolated mitochondria from cultured cells. (A-B) Isolated mitochondria from MCF10A and MCF7 cells were incubated with either ddH2O(control) or metformin (10 mM) for 30 minutes in a 37°C water bath in the presence of complex I substrates (malate and pyruvate). Respiration was tested immediately (State 2), followed by the addition of ADP (State 3) and oligomycin (State 4), as indicated by the arrows above the trace. (C-D) Fold change values represent fold change from incubated but untreated mitochondria. N = 3, *P <0.05, Student’s t-test. Figure S3. is related to Figure 4; Metformin decreases respiration in a time-dependent manner in isolated mitochondria. Isolated mitochondria from murine skeletal muscle were incubated in a 37°C respiration chamber in the presence of complex I substrates: malate, pyruvate as well as oligomycin, (state 4) with the addition of either ddH2O (control) or metformin (10 mM) and recorded immediately at t = 0 (A). (B) The changes in respiration at the end of the recording period (t = 20 min) are represented as fold change values. Experiments were repeated for state 2 conditions (malate and pyruvate) (C) and fold change values (D) were calculated from incubated but untreated mitochondria. N = 3, *P <0.05, Student’s t-test. [file 2049-3002-2-12-S2.pdf]
